# Circulating high mobility group box-1 and toll-like receptor 4 expressions increase the risk and severity of epilepsy

**DOI:** 10.1590/1414-431X20197374

**Published:** 2019-06-19

**Authors:** Minchen Kan, Lihong Song, Xueqiang Zhang, Jing Zhang, Pingping Fang

**Affiliations:** 1 Department of Neurology, HanDan Central Hospital, Handan, China; 2 Medical Department, HanDan Central Hospital, Handan, China; 3 HanDan Central Hospital, Handan, China

**Keywords:** High-mobility group box-1 (HMGB1), Toll-like receptor 4 (TLR4), Epilepsy, Severity, Drug resistance

## Abstract

This study aimed to investigate the association of serum high-mobility group box-1 (HMGB1) and toll-like receptor 4 (TLR4) expressions with the risk of epilepsy as well as their correlations with disease severity and resistance to anti-epilepsy drugs. One hundred and five epilepsy patients and 100 healthy controls (HCs) were enrolled in this case-control study, and serum samples were collected from all participants to assess the HMGB1 and TLR4 expressions by enzyme-linked immunosorbent assay (ELISA). Both serum HMGB1 (P<0.001) and TLR4 (P<0.001) expressions were higher in epilepsy patients than in HCs, and they displayed good predictive values for risk of epilepsy. Moreover, HMGB1 was positively correlated with TLR4 level (r=0.735, P<0.001). HMGB1 and TLR4 levels were both elevated in patients with an average seizure duration >5 min compared to patients with a seizure duration ≤5 min (P=0.001 and P=0.014, respectively). Also, HMGB1 and TLR4 were increased in patients with seizure frequency >3 times per month compared to patients with seizure frequency ≤3 times per month (both P=0.001). In addition, HMGB1 and TLR4 expressions were higher in intractable cases compared to drug-responsive cases (P<0.001). In conclusion, both HMGB1 and TLR4 expressions were correlated with increased risk and severity of epilepsy and their level was higher in patients resistant to anti-epilepsy drugs.

## Introduction

Epilepsy, which is characterized by unprovoked and recurrent seizures, is a common brain disorder associated with elevated mortality rate, decreased social participation, as well as declined quality of life (1–3). The pathogenesis of epilepsy is associated with massive alterations in the expression of genes that control neurotransmitter signaling, ion channels, synaptic structure, neuronal death, gliosis, and inflammation (3). The disorder affects more than 50 million people worldwide, and an additional 2.4 million new cases of epilepsy are diagnosed every year (4,5). Although around 20 antiepileptic drugs are available for epilepsy patients, 30% of the patients still present uncontrolled seizures (4). Thus, comprehensive and precise understanding of the molecular mechanisms underlying seizures and efforts to develop new approaches for treatment are necessary.

Mounting evidence suggests that inflammation resulting from stroke, infection, neurotrauma, etc is correlated with the occurrence of epilepsy. Moreover, it is reported that some inflammatory mediators such as cytokines, complement factors, and prostaglandins play roles in epileptogenesis (6–9). Toll-like receptors (TLRs) are proteins that are fundamental in the induction of the immune system and inflammatory reaction. Among the members of the TLRs family, toll-like receptor 4 (TLR4), which is a lipopolysaccharide-sensing receptor that triggers inflammation by inducing gene transcription, has attracted great attention in various diseases (10). High-mobility group box-1 (HMGB1), an endogenous ligand of TLR4 and one of the damage-associated molecular patterns (DAMPs), is released from injured tissues (11,12). Studies have revealed that both HMGB1 and TLR4 contribute to the occurrence and persistence of seizures (13–15). For instance, HMGB1 is responsible for the unfavorable inflammatory effects in epilepsy. As to TLR4, the resistance of kainite-induced seizures has been observed in TLR4 knockout mice (13–17). However, most of these previous studies investigating the function of HMGB1 and TLR4 in epilepsy were performed in experimental animal models or brain specimens; few clinical studies have been performed about the roles of HMGB1 and TLR4 in epilepsy patients (13–15). Furthermore, few studies investigated the effects of HMGB1 and TLR4 on drug resistance in patients with epilepsy (18). Therefore, we conducted this case-control study to investigate the association of serum HMGB1 and TLR4 expressions with the risk of epilepsy as well as their correlations with disease severity and anti-epilepsy drugs resistance.

## Material and Methods

### Participants

This case-control study enrolled 105 epilepsy patients and 100 healthy controls (HCs) in HanDan Central Hospital from January 2015 to December 2016. The inclusion criteria of epilepsy patients were as follows: i) diagnosed as epileptic according to 2010 International League Against Epilepsy (ILAE) classification, ii) at least two well documented and unprovoked seizures that were clinically evaluated and classified as partial or generalized seizures, and iii) documented electroencephalogram result. Exclusion criteria of epilepsy patients were as follows: 1) secondary epilepsy originated from craniocerebral injury, cerebral hemorrhage, cerebral infarction, intracranial space-occupying lesions, and inherited metabolic diseases, ii) receiving glucocorticoid or immunosuppressive agent treatments, and iii) recent moderate to severe infection. HC participants with a malignant solid tumor or malignant hematological disease history, severe infection history, severe renal or hepatic dysfunction history, brain injury or related diseases, cognitive impairment, and receiving glucocorticoid or immunosuppressive agent treatments were excluded.

Both epilepsy patients and HCs or their statutory guardian provided written informed consent, and the Ethics Committee of HanDan Central Hospital approved this study.

### Data collection and definitions

Data of epilepsy patients including age, gender, disease status (generalized or partial), disease course, average seizure duration, seizure frequency, and drug resistance status were collected after enrollment. Data of HCs were recorded after recruitment including age and gender. Drug resistance status was classified as follows: intractable epilepsy, which was defined as seizure occurrence after two or more doses of first-line anti-epilepsy drug of reasonable concentrations were given daily for at least 2 years. Drug-responsive epilepsy was defined as the opposite of intractable epilepsy. The anti-epilepsy drugs that patients received are reported in Supplementary Table S1.

### Sample collection

Two milliliters fasting peripheral venous blood samples were collected from epilepsy patients within 12 h after the seizure. Meanwhile, 2 mL fasting peripheral venous blood samples were obtained from healthy participants. Subsequently, the blood samples were centrifuged for 20 min at 625 *g* (25^o^C) to get serum after 30 min standing at room temperature. Then, the collected serum samples were stored at -80°C in the refrigerator.

### HMGB1 and TLR4 detection

The expressions of HMGB1 and TLR4 were assessed by a commercial ELISA kit (Sangon Biotech, China) following the instructions of the manufacturer.

### Statistical analysis

Statistical analysis was performed using SPSS 21.0 software (IBM, USA) and Graphpad Prism 5.01 software (GraphPad Software Inc., USA). Data are reported as median (25th–75th percentile), means±SD, or count (%). Comparison between two groups was determined by the Mann-Whitney test, *t*-test, or chi-squared test. Receiver operating characteristic (ROC) curves were performed to evaluate the predictive values of HMGB1 and TLR4 for epilepsy risk. Spearman’s test was conducted to analyze the correlation between HMGB1 and TLR4. A P value <0.05 was considered significant.

## Results

### Baseline characteristics

General characteristics of epilepsy patients and HCs are shown in Table 1.

**Table 1.** Baseline characteristics.

Parameters	Epilepsy patients (n=105)	Healthy controls (n=100)	P value
Age (years, means±SD)	29.65±10.82	29.83±10.51	0.903
Gender (male/female)	61/44	48/52	0.380
Disease status (n, %)			
Generalized epilepsy	31 (29.5)		
Partial epilepsy	74 (70.5)		
Disease course (n, %)			
>2 years	66 (62.9)		
≤2 years	39 (37.1)		
Seizure duration (n, %)			
>5 min average	54 (51.4)		
≤5 min average	51 (48.6)		
Seizure frequency (n, %)			
>3 times per month	29 (27.6)		
≤3 times per month	76 (72.4)		
Drug resistance status (n, %)			
Intractable epilepsy	51 (49.0)		
Drug-responsive epilepsy	54 (51.0)		

Comparison between two groups was determined by the *t*-test or chi-squared test. P<0.05 was considered significant.

### HMGB1 and TLR4 expressions in epilepsy patients and HCs

**Figure 1.**
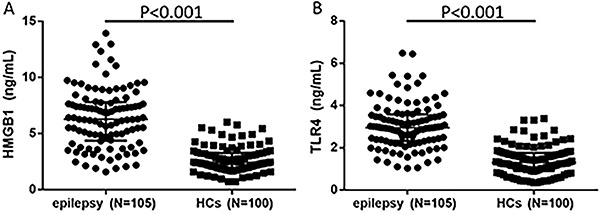
**A**, Expression of HMGB1 in epilepsy and healthy control (HCs) groups. **B**, Expression of TLR4 in epilepsy and HCs groups. TLR4, toll-like receptor 4; HMGB1, high-mobility group box-1. Statistical analysis by Mann-Whitney test.

Expressions of both HMGB1 (6.3, 4.4−7.8 ng/mL *vs* 1.9, 2.4−3.3 ng/mL, P<0.001) and TLR4 (3.0, 2.3−3.6 ng/mL *vs* 0.9, 1.3−1.7 ng/mL, P<0.001) in epilepsy patients were greatly elevated compared with HCs (Figure 1).

### Predictive values of HMGB1 and TLR4 expressions for risk of epilepsy

**Figure 2.**
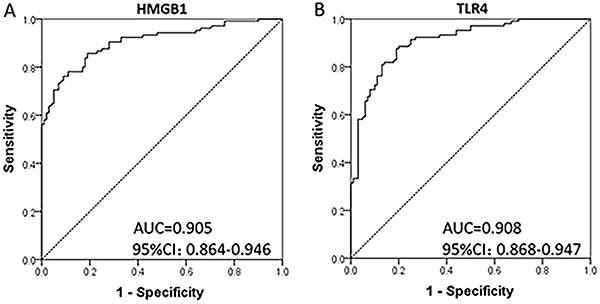
Receiver operating characteristic curves of HMGB1 (**A**) and TLR4 (**B**) for risk of epilepsy. TLR4, toll-like receptor 4; HMGB1, high-mobility group box-1.

HMGB1 displayed a good predictive value of epilepsy risk with an AUC of 0.905 (95%CI, 0.864−0.946). TLR4 also presented a good epilepsy prediction with an AUC of 0.908 (95%CI, 0.868−0.947) (Figure 2).

### Correlation between HMGB1 and TLR4

**Figure 3.**
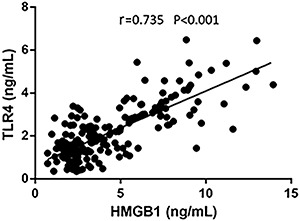
Correlation between HMGB1 and TLR4 determined by Spearman’s test. TLR4, toll-like receptor 4; HMGB1, high-mobility group box-1.

Correlation between HMGB1 and TLR4 expressions was analyzed by Spearman’s test. HMGB1 was positively correlated with TLR4 level (r=0.735, P<0.001) (Figure 3).

### Comparison of HMGB1 among subgroups of epilepsy patients

**Figure 4.**
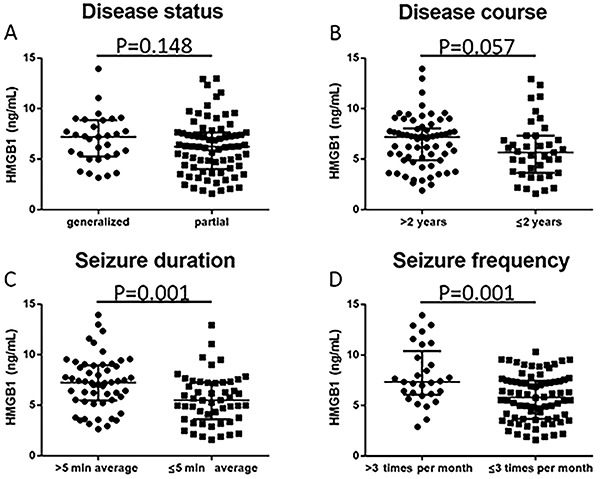
Comparison of high-mobility group box-1 (HMGB1) between subgroups in epilepsy patients using the Mann-Whitney test. **A**, Disease status; **B**, disease course; **C**, seizure duration; **D**, seizure frequency.

To further investigate the effect of HMGB1 in epilepsy patients, we divided patients into different subgroups according to disease status, disease course, seizure duration, and seizure frequency (Figure 4). HMGB1 level was higher in patients with seizure duration >5 min average than patients with seizure duration ≤5 min average (P=0.001). Patients with seizure frequency >3 times per month presented higher HMGB1 expressions compared with patients with seizure frequency ≤3 times per month (P=0.001). Additionally, patients with disease course >2 years displayed higher HMGB1 levels compared with those with disease course ≤2 years but no statistical significance was observed (P=0.057). No difference in HMGB1 expression was found between patients with generalized disease and patients with partial disease (P=0.148).

### Comparison of TLR4 by subgroups in epilepsy patients

**Figure 5.**
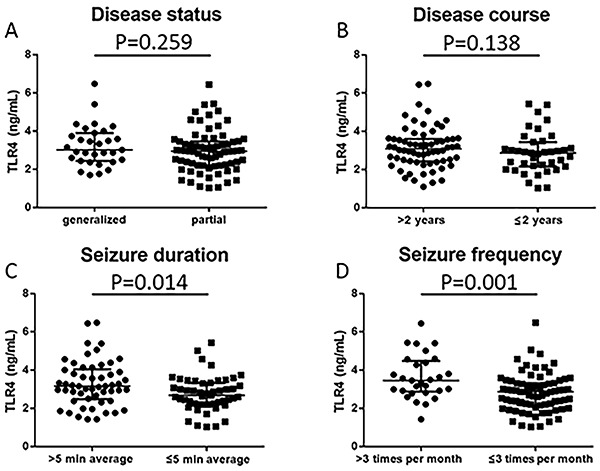
Comparison of toll-like receptor 4 (TLR4) between subgroups in epilepsy patients using the Mann-Whitney test. **A**, Disease status; **B**, disease course; **C**, seizure duration; **D**, seizure frequency.

As shown in Figure 5, patients with seizure duration >5 min average presented with higher TLR4 than patients with seizure duration ≤5 min average (P=0.014). Likewise, patients with seizure frequency >3 times per month had increased TLR4 compared with patients with seizure frequency ≤3 times per month (P=0.001). However, no difference in TLR4 was found in patients with different disease status (P=0.259) or disease course (P=0.138).

### Correlation of HMGB1 and TLR4 expressions with anti-epilepsy drugs resistance

**Figure 6.**
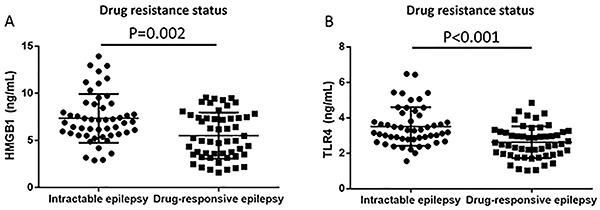
Comparison of HMGB1 (**A**) and TLR4 (**B**) based on drug resistance status using the Mann-Whitney test. TLR4: toll-like receptor 4; HMGB1: high-mobility group box-1.

As shown in Supplementary Table S1, no correlations of HMGB1 and TLR4 expressions with anti-epilepsy drugs were found in epilepsy patients. Epilepsy patients were divided into intractable epilepsy group and drug-responsive epilepsy group. As shown in Figure 6A, HMGB1 expression in patients with intractable epilepsy was significantly higher than that in patients with drug-responsive epilepsy (P=0.002). Moreover, patients with intractable epilepsy had increased TLR4 expression compared with patients with drug-responsive epilepsy (Figure 6B, P<0.001).

## Discussion

We investigated the correlation of serum HMGB1 and TLR4 levels with risk of epilepsy in this case-control study and we found that: i) expressions of HMGB1 and TLR4 were higher in epilepsy patients compared with HCs; ii) elevated HMGB1 and TLR4 expressions were both associated with longer seizure duration as well as increased seizure frequency; iii) increased HMGB1 and TLR4 expressions were correlated with higher possibility of anti-epilepsy drugs resistance.

Factors that induce epileptic seizures including hyperpyrexia, injury, and infection impair astrocytes as well as microglia and subsequently induce the release of HMGB1. The activated glial cells or neurons stimulate the signaling pathway of HMGB1-TLR4, which triggers the release of Ca^2+^ (19–21). Excess Ca^2+^ results in an increase of neuronal excitability and neuron damage, subsequently decreasing seizure threshold, thus more frequent seizures. Based on these mechanisms, HMGB1-TLR4 signaling, as the key point of inflammation in the brain, plays a crucial role in development and progress of epileptic seizures.

Only a few clinical studies have been performed to explore roles of HMGB1 and TRL4 expressions in risk and severity of epilepsy seizures. Maroso et al. (16) conducted a study to examine the expression of HMGB1 and TLR4 both in hippocampi of humans with epilepsy and in an epilepsy mice model. Cytoplasmic HMGB1 staining was increased in human hippocampi specimens from drug-resistant temporal lobe epilepsy (TLE) with hippocampal sclerosis (TLE-HS) compared with control subjects. Moreover, they discovered that seizure frequency increased 2.5-fold with HMGB1 overexpression in the mice model, which was due to the elevated activation of the TLR4-HMGB1 pathway. Iori et al. (15) verified that a higher dose of kainic acid, an agent for inducing epilepsy, was needed to induce epilepsy in TLR4-knockout mice compared to TLR4-wild type, and the times and duration of paroxysmal hippocampal seizures were reduced in TLR4-knockout mice with epilepsy. Pernhorst et al. (22) detected the TLR4 expression in astrocytes of pharmacoresistant patients with TLE (n=26), and they discovered that a higher expression of TLR4 was correlated with higher seizure frequency (r=0.408; P=0.02).

These animal experiments and clinical studies suggested that both HMGB1 and TLR4 expressions are upregulated in epilepsy cases. The possible reasons for the results found in our study are as follows: i) excessive HMGB1 is released owing to brain injury or dysfunction and it leads to elevated HMGB1-TLR4 signaling, which results in increased neuronal excitability; ii) seizure threshold is decreased and easier to reach in the epilepsy cases, thus occurrence of seizure becomes more frequent; iii) proinflammatory cytokines, the release of which is induced by excess HMGB1 and in turn stimulate more release of HMGB1, play key roles in the occurrence and perpetuation of seizures.

Furthermore, we found that HMGB1 and TLR4 expressions were both elevated in patients with intractable epilepsy compared with those in patients with drug-responsive epilepsy, and this might be due to: i) patients with drug resistance had more serious dysfunction, injury or other seizure-inducing factors in the brain, which might exacerbate the aberrant expressions of HMGB1 and TLR4; ii) HMGB1 has various isoforms, and acetylated HMGB1 was only expressed in patients with drug resistance; this might lead to heterogeneity of HMGB1 expressions between patients with intractable epilepsy and drug-responsive epilepsy (19).

Our study had some limitations: i) sample size of epilepsy patients was relatively small, thus the statistical power might be poor; ii) this was a case-control study and thus causality cannot be assumed. Cell and mice experiments are needed to validate the causal relationship.

In conclusion, both increased HMGB1 and TLR4 expressions correlated with higher risk and severity of epilepsy as well as the elevated possibility of anti-epilepsy drug resistance.

## Supplementary Material

Click here to view [pdf].
